# Interaction effect between handedness and CNTNAP2 polymorphism (rs7794745 genotype) on voice-specific frontotemporal activity in healthy individuals: an fMRI study

**DOI:** 10.3389/fnbeh.2015.00087

**Published:** 2015-04-20

**Authors:** Michihiko Koeda, Atsushi Watanabe, Kumiko Tsuda, Miwako Matsumoto, Yumiko Ikeda, Woochan Kim, Amane Tateno, Banyar Than Naing, Hiroyuki Karibe, Takashi Shimada, Hidenori Suzuki, Masato Matsuura, Yoshiro Okubo

**Affiliations:** ^1^Department of Neuropsychiatry, Graduate School of Medicine, Nippon Medical SchoolTokyo, Japan; ^2^Division of Personalized Genetic Medicine, Nippon Medical School HospitalTokyo, Japan; ^3^Department of Biochemistry and Molecular Biology, Graduate School of Medicine, Nippon Medical SchoolTokyo, Japan; ^4^Department of Biofunctional Informatics, Tokyo Medical and Dental UniversityTokyo, Japan; ^5^Department of Pediatric Dentistry, Nippon Dental UniversityTokyo, Japan; ^6^Department of Pharmacology, Graduate School of Medicine, Nippon Medical SchoolTokyo, Japan

**Keywords:** *CNTNAP2*, fMRI, voice, handedness, SNPs, autism, schizophrenia

## Abstract

Recent neuroimaging studies have demonstrated that Contactin-associated protein-like2 (*CNTNAP2*) polymorphisms affect left-hemispheric function of language processing in healthy individuals, but no study has investigated the influence of these polymorphisms on right-hemispheric function involved in human voice perception. Further, although recent reports suggest that determination of handedness is influenced by genetic effect, the interaction effect between handedness and *CNTNAP2* polymorphisms for brain activity in human voice perception and language processing has not been revealed. We aimed to investigate the interaction effect of handedness and *CNTNAP2* polymorphisms in respect to brain function for human voice perception and language processing in healthy individuals. Brain function of 108 healthy volunteers (74 right-handed and 34 non-right-handed) was examined while they were passively listening to reverse sentences (rSEN), identifiable non-vocal sounds (SND), and sentences (SEN). Full factorial design analysis was calculated by using three factors: (1) rs7794745 (A/A or A/T), (2) rs2710102 [G/G or A carrier (A/G and A/A)], and (3) voice-specific response (rSEN or SND). The main effect of rs7794745 (A/A or A/T) was significantly revealed at the right middle frontal gyrus (MFG) and bilateral superior temporal gyrus (STG). This result suggests that rs7794745 genotype affects voice-specific brain function. Furthermore, interaction effect was significantly observed among MFG-STG activations by human voice perception, rs7794745 (A/A or A/T), and handedness. These results suggest that *CNTNAP2* polymorphisms could be one of the important factors in the neural development related to vocal communication and language processing in both right-handed and non-right-handed healthy individuals.

## Introduction

It is believed that most people process language predominantly in the left hemisphere, but the biological and molecular mechanisms are unclear. In respect to brain function, previous neuroimaging studies of language processing in healthy subjects have demonstrated cerebral activation at the left hemispheric frontotempo-parietal cortices by using functional magnetic resonance imaging (fMRI) (Frost et al., 1999; Springer et al., 1999; Price, 2000; Koeda et al., 2006a, 2007). On the other hand, recent genetic neuroimaging studies have verified the influence of single nucleotide polymorphisms (SNPs) in relation to brain structure and brain function (Camara et al., 2010; Frielingsdorf et al., 2010; Cuenco et al., 2011; Chen et al., 2012; Forbes et al., 2012; Hajek et al., 2012; Blasi et al., 2013; Clemm Von Hohenberg et al., 2013).

The contactin-associated protein-like 2 (*CNTNAP2*) gene is known as a transcriptional factor regulated by forkhead box P2 (*FOXP2*) gene related to language processing (Grigorenko, 2009; Newbury and Monaco, 2010; Catani et al., 2011; Penagarikano and Geschwind, 2012; Graham and Fisher, 2013; Rodenas-Cuadrado et al., 2014). Recent studies have reported that *CNTNAP2* is associated with human brain development as cell adhesion molecules (Ip et al., 2011; Huang et al., 2013; Muntané et al., 2014). Two studies have shown that genotypes of *CNTNAP2* affect brain function in healthy subjects (Whalley et al., 2011; Kos et al., 2012). One study has demonstrated that the group with A/T genotype in rs7794745, one of the SNPs in *CNTNAP2*, shows significantly greater activation in the right inferior frontal gyrus (IFG) and right temporal lobe compared with the group with A/A genotype during verbal fluency task (Whalley et al., 2011). Another study of event-related potential (ERP) has demonstrated that the waveform in P600 changes in the A/T genotype group in rs7794745 compared with the A/A genotype group (Kos et al., 2012).

Recent reports have indicated that *CNTNAP2* polymorphisms affect brain function for language processing in neurodevelopmental disorders as well as in healthy subjects. *CNTNAP2* has been reported to be an important genetic factor for differentiating the pathogenesis of language impairment in autism spectrum disorder (ASD) or attention-deficit hyperactivity disorder (ADHD) (Sizoo et al., 2010). A study has shown that A/T in rs7794745 of *CNTNAP2* is a risk genotype of autism compared with A/A (Li et al., 2010). The risk allele of *CNTNAP2* is closely associated with reduced white matter volume in ASD, and with a reduction of fractal anisotropy in the cerebellum and frontotemporal cortex (Tan et al., 2010). Further, previous studies have reported that rs2710102 of *CNTNAP2* is associated with language acquisition in early language development (Whitehouse et al., 2011), or language development disorder (Alarcon et al., 2008; Vernes et al., 2008).

Language is processed predominantly in the left hemisphere in most people. According to previous reports, about 95% of right-handed (RH) subjects are left hemispheric dominant (Binder et al., 1997; Springer et al., 1999). In contrast, about 75% of non-right-handed (non-RH) subjects are left hemispheric dominant (Pujol et al., 1999; Szaflarski et al., 2002). This rate of language being processed in the left hemisphere is significantly less in non-RH subjects than in RH subjects. Further, in non-RH subjects, the rate of predominant left hemispheric language dominance with a family history of non-RH subjects is significantly less than that without such a family history (Szaflarski et al., 2002; Liu et al., 2009). These results suggest that genetic effect may affect acquisition of handedness in the stage of language development.

Recent research has reported that SNPs on several genes related to language development affect brain volumes or brain function (Geschwind et al., 2002; Medland et al., 2006; Sun and Walsh, 2006). Especially, there is evidence of SNPs on *CNTNAP2* associated with brain function for language comprehension (Whalley et al., 2011). However, to our knowledge, no study has investigated the interaction effect between handedness and SNPs on *CNTNAP2* for brain activity in language processing. Further, it is unclear whether *CNTNAP2* affects brain function in human voice perception as well as in language processing. Recent neuroimaging studies demonstrated predominantly right hemispheric activation at the bilateral superior temporal gyrus (STG) during passive listening to human voice (Belin et al., 2000; Fecteau et al., 2004; Koeda et al., 2006a; Charest et al., 2013). In addition, studies of patients with autism and schizophrenia have revealed impairment of brain function at the right STG during human voice perception (Ocklenburg et al., 2013). Based on these findings, it seems important to verify the genetic influence on brain function during listening to human voice as well as language, although, to our knowledge, no such study has been documented. Additionally, recent neuroimaging studies have demonstrated that language dominance in non-RH healthy people is different from RH subjects (Szaflarski et al., 2002; Greve et al., 2013; Perlaki et al., 2013), but the genetic influence on handedness and brain function during auditory processing such as language, human voice, and environmental sounds remains unclear.

We aimed to (1) investigate the phenotypic influence of the genotype of *CNTNAP2* in order to verify the cerebral response to human voice perception and lexical-semantic processing in language processing by using fMRI, and (2) clarify whether brain function of language dominance and human voice perception is affected by genetic factor(s) and handedness.

In this study, to clarify the specific polymorphism(s) related to language processing and human voice perception, 2 SNPs (rs7794745 and rs2710102) in *CNTNAP2* were selected. These SNPs are known as biological high-risk markers for ASD, epilepsy, mental retardation, schizophrenia, and cognitive impairment (Friedman et al., 2008; Li et al., 2010; Stein et al., 2011; Clemm Von Hohenberg et al., 2013; Ji et al., 2013; Sampath et al., 2013). We investigated whether these 2 SNPs affect brain function in healthy individuals.

## Materials and methods

### Subjects of fMRI study

One hundred and eight healthy subjects (53 males and 55 females, mean age 26.3 years, *SD* = 6.9) participated in the present study. All 108 volunteers were native speakers of Japanese. None of the control subjects was taking alcohol or medication at the time, nor did they have a history of psychiatric disorder, significant physical illness, head injury, neurological disorder, or alcohol or drug dependence based on the contents of the Japanese version of Diagnostic Interview for Genetic Studies (DIGS). After complete explanation of the study, written informed consent was obtained from all subjects. The study protocol was approved by the Gene Institutional Review Board of Nippon Medical School. The mean period of education (mean ± SD) was 15.7 ± 0.7 years (male: 15.9 ± 0.7 years; female: 15.6 ± 0.7 years). According to data from the UNESCO Institute for Statistics (http://hdr.undp.org/en/data), the expected years of schooling in 2011 in Japan were as follows: male: 15.4; female: 15.1, respectively. The results of one-sample *t*-test for these expected years of schooling were as follows: male [*t*_(52)_ = 5.08, *p* < 0.001]; female [*t*_(54)_ = 5.21, *p* < 0.001], respectively. Based on the Edinburgh Handedness Inventory (EHI) (Oldfield, 1971) and according to the definition of previous studies (Oldfield, 1971; Springer et al., 1999; Szaflarski et al., 2002; Koeda et al., 2013), handedness of RH subjects was defined as equal to or more than 50 in the score of EHI, whereas that of non-RH subjects was defined as less than 50. Accordingly, handedness was 74 RH and 34 non-RH according to EHI (Oldfield, 1971). Mean (±SD) EHI in the 74 RH subjects was 88.5 ± 11.1, and that of the 34 non-RH subjects was −39.6 ± 38.3.

### Sample collection, preparation, and genotyping

Genomic DNA samples were extracted from peripheral blood using standard procedures. Genotype screening for each SNP was performed by small amplicon genotyping (SAG) method based on high-resolution melting curve analysis (Watanabe et al., 2011). *Two* SNPs, rs7794745 located at intron2 and rs2710102 located at intron13 in *CNTNAP2*, were examined. PCR primers were designed to flank the one base pair just adjacent to the target SNP: 5′-GCAGGACCTGGAAAGGCCTAA-3′ (forward), 5′-GGCCTTTGACACTTAGTCTTATCA-3′ (reverse) in rs7794745 and 5′-GGGCCTTTGTTTTTCCTTCTTTCTC-3′ (forward), 5′-GCGGTTAACATTTACTCTGAGACC-3′ (reverse) in rs2710102. We added external GC at the 5′ end of each primer to adjust the GC percentage (shown with underlines). All primers were designed with the LightScanner Primer Design software program (Idaho Technology, UT, USA). DNA amplification was performed with a 96-well plate at a 10-μl final volume containing 4 μl of 2.5× high-sensitivity genotyping master mix (Idaho Technology), 1 μM of each primer, and 20 ng of genomic DNA. The thermocycling conditions were: 2 min at 95°C, followed by 45 cycles of 30 s at 94°C and 30 s at 67°C in a CFX96 Real-Time PCR detection system (Bio-Rad Laboratories, CA, USA). After PCR, high-resolution melting was performed with a 96-well plate LightScanner (Idaho Technology), which collected data from 55°C to 98°C at a ramp rate of 0.10°C/s. The genotyping of all subjects was determined in comparison with control DNA confirmed by sequencing in the SNP pattern. Genotype of rs7794745 is A/A, A/T, and T/T; that of rs2710102 is G/G, G/A, A/A. In our study, the dominant DNA sequence was presumed to be T in rs7794745 and G in rs2710102 according to the Japanese Hapmap ratio (Table 1). The genotypes were classified into A/A and A/T in rs7794745, and G/G and A carriers (G/A and A/A) in rs2710102, respectively. In rs2710102, the genotypes of C/C, C/T, and T/T were used in some studies (Whalley et al., 2011; Whitehouse et al., 2011; Zhou et al., 2012; Clemm Von Hohenberg et al., 2013; Sampath et al., 2013), and the genotypes of G/G, G/A, and A/A in an opposite strand were used in other studies (Stein et al., 2011; Ji et al., 2013). In this study, we used the genotypes of G/G, G/A, and A/A in rs2710102.

**Table 1:** **Baseline characteristics of subjects**.

Total subject number	105			
M/F	52/53			
Handedness (non-RH/RH)	34/71			
EHI	RH: 88.5 ± 11.1			
	non-RH: −39.6 ± 38.3			
LFH (Y/N)	39/66			
**rs7794745**	**A/A**	**A/T**		***p*-value**
*n*	57	48		–
M/F	27/30	25/23	*X*^2^ = 0.23	n.s.
Age	26.2 ± 6.8	26.2 ± 7.0	*t*_(103)_ = 0.03	n.s.
Handedness (non-RH/RH)	19/38	15/33	*X*^2^ = 0.05	n.s.
EHI	RH: 87.5 ± 12.5	RH: 90.2 ± 9.4	*t*_(69)_ = −1.04	n.s.
	non-RH: −35.2 ± 39.1	non-RH: −45.1 ± 37.9	*t*_(32)_ = 0.74	n.s.
LFH (Y/N)	19/38	20/28	*X*^2^ = 0.78	n.s.
Education (years)	15.7 ± 0.7	15.7 ± 0.6	*t*_(103)_ = 0.24	n.s.
Hapmap (ss44810024) [ratio (%)]/our current study [ratio (%)]	43/54.3	57/45.7	*X*^2^ = 2.42	n.s.
**rs2710102**	**G/G**	**A carriers (G/A and A/A)**		***p*-value**
*n*	54	51		–
M/F	29/25	23/28	*X*^2^ = 0.78	n.s.
Age	25.9 ± 6.5	26.6 ± 7.3	*t*_(103)_ = −0.55	n.s.
Handedness (non-RH/RH)	20/34	14/37	*X*^2^ = 1.10	n.s.
EHI	RH: 89.3 ± 11.0	RH: 88.3 ± 11.5	*t*_(69)_ = 0.37	n.s.
	non-RH: −43.3 ± 39.0	non-RH: −34.3 ± 38.1	*t*_(32)_ = −0.66	n.s.
LFH (Y/N)	23/31	16/35		n.s.
Education (years)	15.7 ± 0.6	15.7 ± 0.7	*t*_(103)_ = −0.31	n.s.
Hapmap (ss44810024) [ratio (%)]/our current study [ratio (%)]	55.3/51.4	44.7/48.6	*X*^2^ = 0.32	n.s.

*Characteristics of two SNPs (rs7794745 and rs2710102) of CNTNAP2. Bottom line: comparison of allele frequency between international HapMap Project (http://www.ncbi.nlm.nih.gov/projects/SNP/) and our current study. Abbreviations: n, number of subjects; M/F, males/females; RH, right-handed subjects; non-RH, non-right-handed subjects; EHI, Edinburgh Handedness Inventory; LFH, left-handed family history (LFH)*.

### Experiment design of fMRI

**Figure 1:**
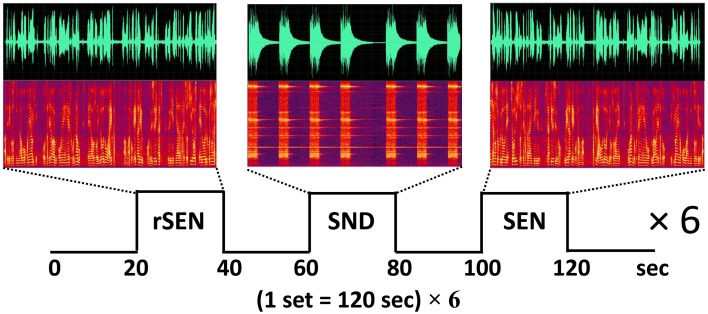
**Protocol of fMRI experiment**. The subjects passively listened to reverse sentences (rSEN), identifiable non-vocal sounds (SND), and sentences (SEN) during MRI scanning. The top row shows the time-domain waveforms, the horizontal axis shows the tone domain, and the vertical axis shows the power of the sound. The middle row shows the sound spectrograms under the three sound conditions. The horizontal axis shows the time domain, and the vertical axis shows the frequency (about 0–4000 Hz) of the tone domain.

Based on a previously published fMRI protocol (Koeda et al., 2006a,b, 2007), passive listening fMRI experiments were performed (Figure 1). The subjects listened to three types of stimuli: forward-played sentences (SEN); the same sentences, but played in reverse (rSEN); and identifiable nonvocal sounds (SND). The duration of each stimulus (SEN, rSEN, and SND) was 20 s. The subjects listened to these 3 stimuli, each followed by a silence of 20 s, alternately. In our fMRI study, a set of the experiment, silence-stimulus-silence-stimulus-silence-stimulus, was repeated six times. These three stimuli (rSEN, SND, SEN) were played pseudo-randomly in each set of the experiment (Figure 1). Contents of SEN stimuli used are shown in Appendix 1. Total scanning time was 720 s (120 s × 6). After the subjects were scanned by fMRI, they answered a questionnaire in order to confirm the performance based on previous studies (Koeda et al., 2006a,b, 2007). Regarding rSEN, the subjects were asked about the type of sounds (i.e., human or non-human), gender of sounds, and whether these sounds included intonation or meaning (See Appendix 2). Questions of Appendix 2 were asked after the subject listened to rSEN stimuli. As for SND, the subjects were asked what kind of sounds they listened to. Concerning SEN, the subjects were asked several questions regarding the contents of each story in multiple choice format.

### Instruments used for presentation of stimuli

Stimuli were presented by the use of Media Studio Pro (version 6.0 Ulead Systems, Inc., Taiwan) running under Windows XP. Subjects listened to the sound stimuli through headphones attached to an air conductance sound delivery system. The average sound pressure of stimulus amplitude was kept at 80 dB.

### fMRI acquisition

Images were acquired with a Phillips 3.0 Tesla scanner. Functional images of 395 volumes were acquired with T2^*^-weighted gradient echo planar imaging sequences sensitive to blood oxygenation level-dependent (BOLD) contrast. Each volume consisted of 35 transaxial contiguous slices, slice thickness 4 mm, to cover almost the whole brain (flip angle, 72.5°; time to echo [TE], 23 ms; repetition time [TR], 1.6 s; matrix, 52 × 30 × 64; field of view, 208 × 120 × 256).

### Image processing

Data analysis was performed with statistical parametric mapping software SPM8 (Wellcome Department of Cognitive Neurology, London, United Kingdom) running with MATLAB (Mathworks, Natick, MA). All volumes of functional EPI images were realigned to the first volume of each session to correct for subject motion. These images were spatially normalized to the standard space defined by Montreal Neurological Institute (MNI) template. After normalization, all scans had a final resolution of 2 × 2 × 2 mm^3^. Functional images were spatially smoothed with a 3-D isotropic Gaussian kernel (full width at half maximum of 8 mm). Low-frequency noise was removed by applying a high-pass filter temporal smoothing function to the fMRI time series to enhance the temporal signal-to-noise ratio. The significance of hemodynamic changes in each condition was examined using the general linear model with boxcar functions convoluted with a hemodynamic response function. Statistical parametric maps for each contrast of the t-statistics were calculated on a voxel-by-voxel basis. The *t*-values were then transformed to unit normal distribution, resulting in z-scores. Data were excluded if the motion artifact was more than 2 mm on the head location (x, y, z translation; pitch, roll, and yaw) at the stage of realignment based on a previous study (Christodoulou et al., 2013).

The models of 3 contrasts (rSEN, SND, and SEN) were created by blocked design during the fMRI experiments (Figure 1). At first, investigating the SNP effect on cerebral activation in auditory processing, cerebral activation under the 3 contrasts was analyzed.

Next, based on our previous studies (Koeda et al., 2006a,b, 2007), to clarify cerebral activation in human voice perception, cerebral activation under rSEN minus SND was examined. Further, cerebral activation under SEN minus rSEN was examined to verify the effect of cerebral activation on language processing (Figure 1).

### Statistical analysis

Group analysis (2nd-level analysis in SPM8) was performed on the data of the 108 control subjects using a random effect model on a voxel-by-voxel basis. First, in order to examine the effects of SNPs on cerebral activation in general auditory processing, fMRI data were analyzed based on the 2 × 2 × 3 full factorial model with the factors of A carriers (A/A or A/T) in rs2710102, [G/G, or A carriers (G/A and A/A)] in rs2710102, and task (rSEN or SND or SEN) [voxel level: *p* < 0.001, cluster level *p* < 0.05 corrected for multiple comparisons, Monte–Carlo simulation (*n* = 1000)]. This statistical threshold was determined based on a previous fMRI study (Slotnick et al., 2003). Second, in order to investigate the effects of SNPs on the cerebral activation of human voice perception, fMRI data were analyzed based on the 2 × 2 × 2 full factorial model with the factors of task (rSEN or SND), rs7794745 (A/A or A/T) and rs2710102 [G/G, or A carriers (G/A and A/A)], [voxel level: *p* < 0.005, cluster level *p* < 0.05 corrected for multiple comparisons, Monte–Carlo simulation (*n* = 1000)]. Third, to investigate the effects of SNPs in *CNTNAP2* on cerebral activation of language processing, fMRI data were analyzed based on the 2 × 2 × 2 full factorial model with the factors of task (rSEN or SEN), A carriers (A/A or A/T) in rs7794745 and G/G, or A carriers (G/A and A/A) in rs2710102 [voxel level: *p* < 0.005, cluster level *p* < 0.05 corrected for multiple comparisons, Monte–Carlo simulation (*n* = 1000)]. By using rfxplot (Glascher, 2009), cerebral activation at the regions of interests (ROIs) was investigated. In the main effect of task, ROIs were focused on sphere voxels of 10 mm radius from the coordinates of the peak voxel of activation.

## Results

### Baseline characteristics of subjects

The fMRI data of 3 subjects were excluded due to motion artifacts, and thus the data of 105 subjects were analyzed in this study. Table 1 shows the baseline characteristics of the subjects. Among the 105 subjects, 57 subjects possessed A/A genotype in rs7794745, and 48 possessed A/T genotype, whereas in rs2710102, 54 possessed G/G genotype, 41 G/A genotype, and 10 A/A genotype. Finally, in the current study, since A/A genotype in rs2710102 was seen in only a few subjects, two phenotypes were compared: (1) G/G in rs2710102, and (2) A carrier (G/A and A/A) in rs2710102.

For each genotype of both rs7794745 and rs2710102, the rates of gender (M/F), age, handedness, EHI, LFH, and education are summarized in Table 1. No significant differences were observed in these factors between rs7794745 genotypes (A/A or A/T), and between rs2710102 genotypes [G/G or A carrier (G/A, and A/A)] (Table 1). Further, the genotype frequency of the known rate of Japanese, based on the database of the international HapMap Project (http://hapmap.ncbi.nlm.nih.gov/), was compared with the subjects' rates in the current study (Table 1). The Hapmap Japanese rate was examined by using the following websites: rs7794745: http://www.ncbi.nlm.nih.gov/projects/SNP/snp_ref.cgi?rs=7794745; rs2710102: http://www.ncbi.nlm.nih.gov/projects/SNP/snp_ref.cgi?rs=2710102. NCBI Assay IDs in this project were rs7794745: ss44810024; rs2710102: ss11865581, respectively. No significant difference was observed between the HapMap Japanese rate and our subjects (rs7794745: χ^2^ = 2.42, *p* > 0.05; rs2710102: χ^2^ = 0.32, *p* > 0.05).

### Behavioral data (accuracy)

**Figure 2:**
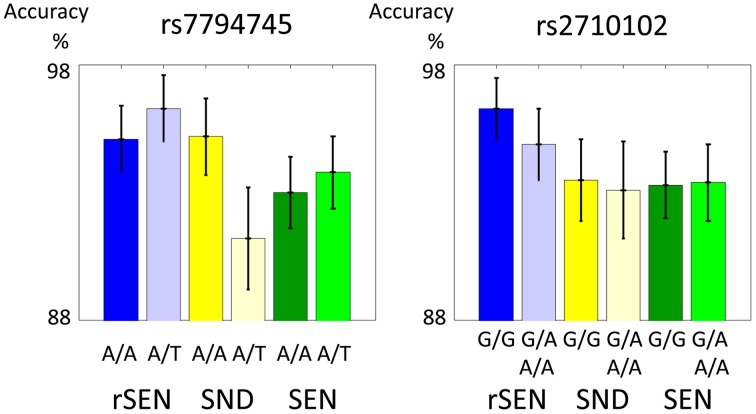
**Performance during fMRI experiments**. These two figures show the distribution of fMRI performance in rs7794745 (left side) and rs2710102 (right side) of *CNTNAP2* gene. In both SNPs, there were no significant differences in the performance of rSEN, SND, and SEN (*p* > 0.05). (1) rSEN: rs7794745 A/A: blue bar, rs7794745 A/T: light blue bar, (2) SND: rs7794745 A/A: yellow bar, rs7794745 A/T: light yellow bar, (3) SEN: rs7794745 A/A: green bar, rs7794745 A/T: light green bar, (4) rSEN: rs2710102 G/G: blue bar, rs2710102 G/A and A/A: light blue bar, (5) SND: rs2710102 G/G: yellow bar, rs2710102 G/A and A/A: light yellow bar, (6) SEN: rs2710102 G/G: green bar, rs2710102 G/A and A/A: light green bar.

In the fMRI experiment, the mean percentages (±SD) of accuracy of rs7794745 for rSEN, SND, and SEN were as follows: [rs7794745 A/A] rSEN: 95.1 ± 1.3, SND: 95.2 ± 1.5, SEN 93.0 ± 1.4, [rs7794745 A/T] rSEN: 96.3 ± 1.3, SND: 91.2 ± 2.0, SEN: 93.8 ± 1.4, [rs2710102 G/G] rSEN: 96.3 ± 1.2, SND: 93.5 ± 1.6, SEN: 93.3 ± 1.3, [rs2710102 A carriers (G/A and A/A)] rSEN: 94.9 ± 1.4, SND 93.1 ± 1.9, SEN 93.4 ± 1.5. The 2 × 3 mixed ANOVA was not significantly different from the main effect of rs7794745 [*F*_(1, 103)_ = 0.36, *p* > 0.05; effect size (Partial Eta Squared) < 0.01], main effect of task [*F*_(2, 206)_ = 1.70, *p* > 0.05; effect size (Partial Eta Squared) = 0.02], and interaction effect [*F*_(2, 206)_ = 1.83, *p* > 0.05; effect size (Partial Eta Squared) = 0.02] (Figure 2; Table 2; Supplemental Table 1). Similarly, the 2 × 3 mixed ANOVA was not significantly different from the main effect of rs2710102 [*F*_(1, 103)_ = 0.23, *p* > 0.05; effect size (Partial Eta Squared) < 0.01], main effect of task [*F*_(1, 103)_ = 2.92, *p* > 0.05; effect size (Partial Eta Squared) = 0.03], and interaction effect [*F*_(2, 206)_ = 0.13, *p* > 0.05; effect size (Partial Eta Squared) < 0.01] (Figure 2; Table 2; Supplemental Table 1), respectively.

**Table 2:** **Performance ratio (%)**.

		**rSEN**	**SND**	**SEN**
rs7794745	A/A	95.1 ± 1.3	95.2 ± 1.5	93.0 ± 1.4
	AT	96.3 ± 1.3	91.2 ± 2.0	93.8 ± 1.4
rs2710102	G/G	96.3 ± 1.2	93.5 ± 1.6	93.3 ± 1.3
	G/A and A/A	94.9 ± 1.4	93.1 ± 1.9	93.4 ± 1.5

*Mean (±SE) of performance in each allele of rs7794745 (upper part) and rs2710102 (lower part) during fMRI experiments*.

### fMRI data

#### Full factorial design analysis

**Figure 3:**
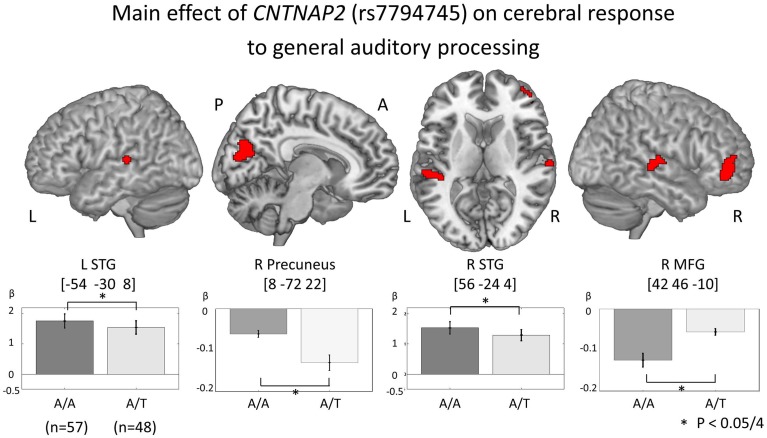
**Main effect of *CNTNAP2* (rs7794745) on cerebral response to general auditory processing**. The results of the main effect of rs7794745 (A/A or A/T) in the analysis of full factorial design are shown (voxel level: *p* < 0.001, cluster level: *p* < 0.05/4, Monte–Carlo simulation). The bars show the distribution of beta values of brain function in general auditory processing at the MNI coordinates of peak activation (A/A: gray, rs7794745 A/T: light gray). Asterisk (^*^) shows a significant rs7794745 effect (A/A and A/T) with Bonferroni correction in 4 ROIs (*p* < 0.05/4). Abbreviations: L, left; R, right; A, anterior; P, posterior.

In order to investigate genetic effects on cerebral activation in general auditory processing, fMRI data was analyzed based on the 2 × 2 × 3 full factorial design with three factors: rs7794745 (A/A or A/T), rs2710102 [G/G or A carriers (G/A and A/A)], and task (rSEN or SND or SEN) [voxel level: *p* < 0.001, cluster level *p* < 0.05 corrected for multiple comparisons, Monte–Carlo simulation (*n* = 1000)]. Main effect of rs7794745 (A/A or A/T) was significantly observed in the bilateral STG, R precuneus, and R MFG (voxel level: *p* < 0.001, cluster level *p* < 0.05 corrected, R: right) (Figure 3 and Table 3A), whereas main effect of rs2710102 [G/G, or A carriers (G/A, and A/A)] was not significantly different. From the results of main effect of rs7794745 on cerebral response to general auditory processing, ROIs were set on 4 regions: L STG [−54, −30, 8], R Precuneus [8, −72, 22], R STG [56, −24, 4], and R MFG [42, 46, −10] (L: left).

**Table 3:** **(A) *CNTNAP2* (rs7794745) effect of cerebral activation of general auditory processing. (B) *CNTNAP2* (rs7794745) effect of cerebral response to human voice perception. (C) *CNTNAP2* (rs7794745) effect of cerebral response to language processing**.

**Brain Regions**	**BA**	**Coordinate**			***F*_(1, 303)_**	***z*-value**	***P* (cluster-level, corrected)**
		***x***	***y***	***z***			
**A**							
**A/A < A/T**							
R MFG	11	42	46	−10	21.85	4.44	<0.001 (<0.05/4)
**A/A > A/T**							
L STG	22	−54	−30	8	17.33	3.94	0.001 (<0.05/4)
R STG	22	56	−24	4	16.78	3.87	0.002 (<0.05/4)
R Precuneus	31	8	−72	22	16.12	3.79	0.002 (<0.05/4)
**B**							
**A/A < A/T**							
R MFG	11	46	48	0	14.18	3.52	0.005 (<0.05/3)
**A/A > A/T**							
R STG	22	56	−24	4	12.58	3.30	0.010 (<0.05/3)
L STG	22	−54	−30	6	11.89	3.20	0.013 (<0.05/3)
**C**							
**A/A > A/T**							
R MFG	11	42	46	−8	11.35	3.12	0.019 (<0.05)

*Note for Table A: Peak coordinates (x, y, z) and their z-values of cerebral activation of general auditory processing with main effect of rs7794745 (A/A or A/T) by full factorial design analysis. Abbreviations: L, left hemisphere; R, right hemisphere; BA, Broadmann's area; MFG, middle frontal gyrus; STG, superior temporal gyrus*.

*Note for Table B: Peak coordinates (x, y, z) and their z-values of cerebral activation of human voice perception with main effect of rs7794745 in full factorial design analysis. Abbreviations: L, left hemisphere; R, right hemisphere; BA, Brodmann's area; MFG, middle frontal gyrus, STG, superior temporal gyrus*.

*Note for Table A: Peak coordinates (x, y, z) and their z-values of cerebral activation of language processing with main effect of rs7794745 in full factorial design analysis. R, right hemisphere; BA, Brodmann's area*.

**Figure 4:**
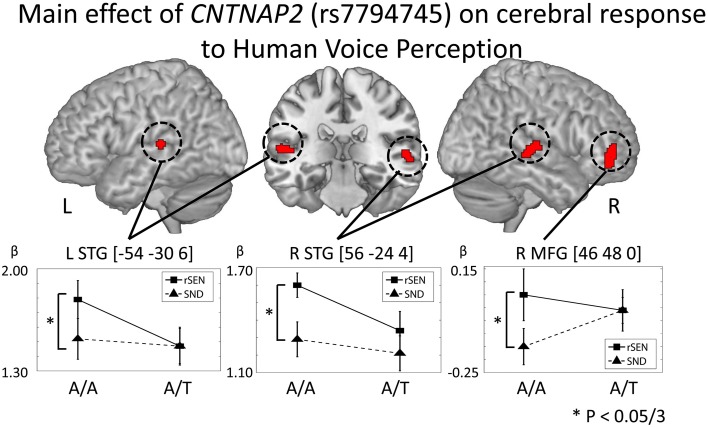
***CNTNAP2* (rs7794745) effect on cerebral response to human voice perception**. The results of the main effect of rs7794745 in the analysis of full factorial design are shown (voxel level: *p* < 0.001, cluster level: *p* < 0.05/3, Monte–Carlo simulation). The error bar shows the distribution of beta values in cerebral activation under rSEN minus baseline (mean: black squares, line) and under SND minus baseline (mean: black triangles, dashed line) at the peak coordinates with the main effect of rs7794745. Asterisks (^*^) show significant differences with Bonferroni correction in the 3 ROIs (*p* < 0.05/3). Abbreviations: L, left hemisphere; R, right hemisphere; MFG, middle frontal gyrus; STG, superior temporal gyrus.

In order to examine the genetic effects on cerebral response to human voice perception, fMRI data were analyzed based on the 2 × 2 × 2 full factorial design with the three factors: rs7794745 (A/A or A/T), rs2710102 [G/G, or A carriers (G/A and A/A)], and task (rSEN or SND) [voxel level: *p* < 0.001, cluster level *p* < 0.05 corrected for multiple comparisons, Monte–Carlo simulation (*n* = 1000)]. Main effect of rs7794745 (A/A or A/T) was significantly observed in bilateral STG and R MFG (voxel level: *p* < 0.005, cluster level *p* < 0.05 corrected) (Figure 4 and Table 3B), whereas main effect of rs2710102 [G/G, or A carriers (G/A and A/A)] was not significantly different. From the results of main effect of rs7794745, ROIs were set on 3 regions: L STG [−54, −30, 6], R STG [56, −24, 4], and R MFG [46, 48, 0]. In these ROIs, beta values under rSEN or SND conditions were calculated by using rfxplot (Glascher, 2009). In Figure 4, cerebral activations between rSEN and SND were compared by Wilcoxon Signed Rank Test. In rs7794745 A/A, the beta values of rSEN were significantly greater than those of SND [L STG: *Z* = −3.01, *p* = 0.002 (*p* < 0.05/3); R STG: *Z* = −4.38, *p* < 0.001 (*p* < 0.05/3); R MFG: −3.33, *p* = 0.001, (*p* < 0.05/3)], whereas in rs7794745 A/T, the beta values were not significantly different between rSEN and SND (L STG: *Z* = −0.39, *p* > 0.05/3; R STG: *Z* = −1.90, *p* > 0.05/3; R MFG: *Z* = −0.17, *p* > 0.05/3).

**Figure 5:**
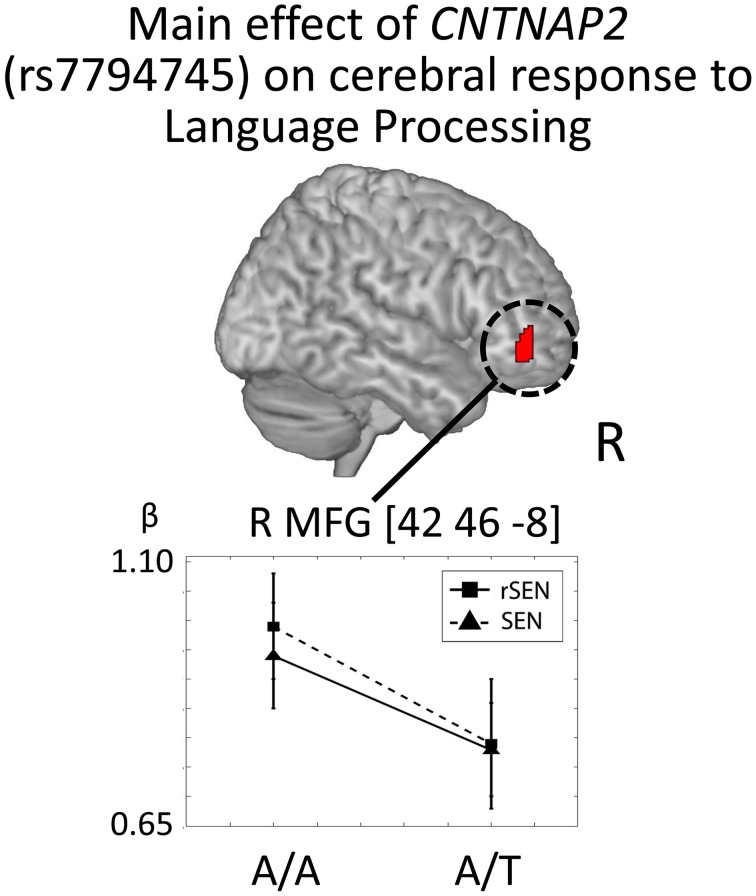
***CNTNAP2* (rs7794745) effect on cerebral response to language processing**. The results of the main effect of rs7794745 (A/A or A/T) in the analysis of full factorial design are shown. The error bar shows the distribution of beta values of cerebral activation under rSEN minus baseline (mean: black squares; dashed line) and under SEN minus baseline (mean: black triangles; line) at the peak coordinates with the main effect of rs7794745 (A/A or A/T). Significant difference was observed in right MFG. Abbreviations: R, right hemisphere; MFG, middle frontal gyrus.

In order to investigate the genetic effects on cerebral activation in language processing, fMRI data were analyzed based on the 2 × 2 × 2 full factorial design with the three factors: rs7794745 (A/A or A/T), rs2710102 [G/G or A carriers (G/A and A/A)], and task (rSEN or SEN) [voxel level: *p* < 0.001, cluster level *p* < 0.05 corrected for multiple comparisons, Monte–Carlo simulation (*n* = 1000)]. Main effect of rs7794745 (A/A or A/T) was significantly observed in R MFG (voxel level: *p* < 0.005, cluster level *p* < 0.05 corrected) (Figure 5 and Table 3C), whereas main effect of rs2710102 [G/G, or A carriers (G/A and A/A)] was not significantly different.

In Figure 5, cerebral activations between rSEN and SEN were compared by Wilcoxon Signed Rank Test. In rs7794745 A/A, the beta value of rSEN was significantly greater than that of SEN [R MFG: *Z* = −4.53, *p* < 0.001 (*p* < 0.05/3)], whereas in rs7794745 A/T, the beta value was not significantly different between rSEN and SEN (R MFG: *Z* = −1.57, *p* > 0.05/3).

**Figure 6:**
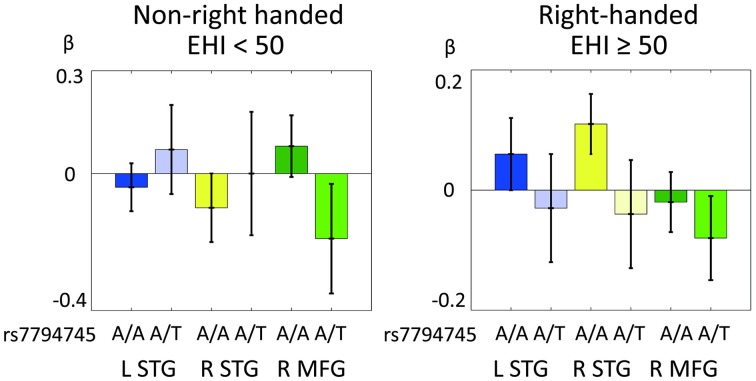
**Comparison of *CNTNAP2* (rs7794745) effect of cerebral response to human voice perception between right-handed (RH) and non-right-handed (non-RH) subjects**. Distributions of BOLD signals of 3 ROIs in human voice perception in non-RH subjects (rs7795745 A/A: *n* = 38; A/T: *n* = 33, left side) and RH subjects (rs7794745 A/A: *n* = 19; A/T: *n* = 14, right side). LTG (A/A: blue, A/T: light blue), STG (A/A: yellow, A/T: light yellow), MFG (A/A: green, A/T: light green), L, left; R, right; EHI, Edinburgh Handedness Inventory.

Figure 6 shows the distribution of cerebral response to human voice perception (cerebral activation under rSEN-SND contrast) in the 3 ROIs (L STG, R STG, and R MFG) in RH and non-RH subjects. In mixed ANOVA [3 ROIs × rs7794745 (A/A or A/T) × LFH × handedness], main effect was not significantly revealed in 3 ROIs, rs7794745 (A/A or A/T), LFH, and handedness (Table 4). Remarkably, interaction effect among the 3 ROIs, rs7794745 (A/A or A/T), and handedness was significantly observed [*F*_(1, 97)_ = 9.94, *p* = 0.002 (*p* < 0.05/3); Table 4], whereas other interaction effects were not significantly observed (Table 4).

**Table 4:** ***CNTNAP2* (rs7794745) effect of cerebral response to human voice perception between RH and non-RH subjects**.

**Mixed ANOVA: 3 ROIs × rs7794745 (A/A or A/T) × LFH × Handedness**		
**Main effect**	***F*-value**	***p***
3 ROIs	*F*_(1.9, 182.4)_ = 1.19	n.s.
rs7794745 (A/A, A/T)	*F*_(1, 97)_ = 0.01	n.s.
LFH	*F*_(1, 97)_ = 1.21	n.s.
Handedness	*F*_(1, 97)_ = 0.97	n.s.
**INTERACTION EFFECT**		
3 ROIs × rs7794745 (A/A, A/T)	*F*_(1, 97)_ = 2.97	n.s.
3 ROIs × LFH	*F*_(1, 97)_ = 1.02	n.s.
3 ROIs × Handedness	*F*_(1, 97)_ = 1.14	n.s.
3 ROIs × rs7794745 (A/A, A/T) × LFH	*F*_(1, 97)_ = 0.33	n.s.
3 ROIs × rs7794745 × Handedness	*F*_(1, 97)_ = 9.94	0.002^*^
3 ROIs × LFH × Handedness	*F*_(1, 97)_ = 0.86	n.s.
3 ROIs × rs7794745 × LFH × Handedness	*F*_(1, 97)_ = 0.67	n.s.

Mixed ANOVA (3 ROIs × rs7794745 × LFH × handedness) was calculated for cerebral activation in human voice perception. Interaction effects among the 3 ROIs, rs7794745 and handedness (EHI < 50, or EHI = 50) were significantly observed. Asterisk (^*^) shows p < 0.05/3.

## Discussion

We aimed to investigate the interaction effect between *CNTNAP2* polymorphism and handedness on linguistic and voice-specific brain activity in healthy individuals. In the current study, the effect of rs7794745 genotype (A/A or A/T) was observed at the bilateral STG, R precuneus, and R MFG on cerebral activation in general auditory processing. Further, the effect of rs7794745 genotype was observed at the bilateral STG and R MFG on cerebral activation in human voice perception, and the effect of rs7794745 genotype was observed at the R MFG on cerebral activation in language processing. Notably, among handedness, rs7794745, and MFG-STG activations by human voice perception, interaction effect was significantly observed. These results suggest that the difference of the specific allele in *CNTNAP2* gene has an influence on brain activity of human voice perception between RH and non-RH subjects.

### The effect of *CNTNAP2* polymorphisms on cerebral response to auditory processing in healthy individuals

In our present results, although the means of the performance ratio were not significantly different between A/A and A/T in rs7794745 (Figure 2 and Table 2), the effect of rs7794745 genotype was significantly observed at the R MFG, bilateral STG, and R precuneus on brain activity to general auditory processing with SEN, rSEN, and SND (Figure 3 and Table 3A). These findings suggest that A/T in rs7794745 of *CNTNAP2* has an influence on the reduction of brain activity in general auditory processing. Further, on brain activity in human voice perception, the effect of rs7794745 genotype was significantly revealed at the bilateral STG and R MFG (Figure 4 and Table 3B). These results indicate that A/T in rs7794745 of *CNTNAP2* affects the decrease in voice-specific brain activity. Recent studies have shown that several SNPs of *CNTNAP2* are biological high-risk markers in ASD, epilepsy, mental retardation, schizophrenia, and cognitive impairment (Friedman et al., 2008; Stein et al., 2011; Li and Bartlett, 2012; Clemm Von Hohenberg et al., 2013; Ji et al., 2013; Sampath et al., 2013). Especially, A/T in rs7794745 was reported to be one of the risks in ASD in the Chinese Han population (Li et al., 2010). In addition, recent neuroimaging studies in healthy subjects demonstrated cerebral activation at the bilateral STG predominantly in the right hemisphere while listening to human voice (Belin et al., 2000; Fecteau et al., 2004; Koeda et al., 2006a; Charest et al., 2013). In contrast, neuroimaging studies in patients with autism showed hypo-activation in R STG (Ocklenburg et al., 2013). However, to our knowledge, no study has investigated the influence of *CNTNAP2* genotype in relation to cerebral response to human voice perception. Our current findings indicate that A/T in rs7794745 of *CNTNAP2*, which is related to neuropsychiatric disorders, affects brain activity of human voice perception in healthy subjects. Voice-specific brain activity is significantly different by the difference of rs7794745 genotype (Figure 4). Voice-specific response was clearly observed in genotype A/A, but it was not revealed in genotype A/T. These findings indicate that A/T in rs7794745 more severely affects the reduction of cerebral response to human voice perception.

### *CNTNAP2* and language processing in the brain

Our results demonstrated the main effect by the difference of rs7794745 genotype at R MFG in lexical-semantic processing of language processing (Figure 5 and Table 3C). A recent neuroimaging study demonstrated increasing brain activity at the right frontotemporal region in language processing by T/T in rs7794745 compared with A carriers (A/A and A/T) (Whalley et al., 2011). On the other hand, our results showed a reduction in right frontal activity of language processing in healthy subjects by rs7794745 A/T compared with A/A. These opposing findings in right frontal activity could be due to differences in the fMRI experiments. The previous fMRI study used a sentence completion paradigm with sentences with the last words missing, asking to silently think of an appropriate word to complete the sentences and press a button when they had done so (Whalley et al., 2011), whereas we investigated brain activity when the subjects were passively listening to contents of the story. However, taking these findings into account, these results suggest that rs7794745 A/T genotype at least affects right hemispheric activity in language processing. In our present study, the effect of rs7794745 genotype was revealed in the right frontal region in language processing despite the lack of significant difference in the performance ratio. In contrast, the effect of rs2710102 genotype was not observed in brain activity of language processing. A recent neuroimaging study reported that, in healthy individuals, rs2710102 is associated with right frontal activity, whereas rs7794745 is related to right temporal activity in language processing (Whalley et al., 2011). In our current study, although rs7794745 genotype of *CNTNAP2* does not influence right temporal activity but rather right frontal activity in language processing, these results do suggest that the genotype of *CNTNAP2* is closely associated with right hemispheric activity in language processing. Recent neuroimaging studies of ASD have shown increasing right frontal activity in language processing (Harris et al., 2006; Knaus et al., 2008; Tesink et al., 2011; Eigsti et al., 2012; Pina-Camacho et al., 2012; Shen et al., 2012). In our current study, the effect of rs7794745 genotype could influence right frontal activity in language processing in healthy subjects. Some neuroimaging studies demonstrated that frontal brain activity in language processing was changed by *FOXP2* gene, which is related to language impairment (Jamadar et al., 2011; Ocklenburg et al., 2013). Taking the previous reports and our current results into consideration, some SNPs of *CNTNAP2* and *FOXP2*, related to language impairment, may directly affect brain activity of language processing as an endophenotype in healthy subjects.

### Influence of *CNTNAP2* on voice-specific brain activity and handedness

We investigated the interaction effect between *CNTNAP2* polymorphism and handedness regarding cerebral response to human voice perception. In mixed ANOVA, interaction effect was revealed between *CNTNAP2* polymorphism [rs7794745 (A/A and A/T)] and handedness regarding the MFG-STG activations to human voice perception (Table 4). Figure 6 shows the distribution of these interaction effects. In our results, the activation in bilateral STG at A/A in rs7794745 was observed as a negative value in non-RH subjects (Figure 6), whereas it was observed as a positive value in RH subjects (Figure 6). In addition, frontal activation at A/A in rs7794745 in non-RH subjects was positive (Figure 6), but that in RH subjects was negative (Figure 6). These results indicate that the activity of MFG-STG by human voice perception shows a differential pattern according to the subject's handedness and type of A/A or A/T in rs7794745 of *CNTNAP2*. On the other hand, the frontal activation in rs7794745 (A/T) showed negative beta values in both RH and non-RH subjects (Figure 6). Further, the left temporal activation at A/T in rs7794745 demonstrated negative beta values in RH subjects (Figure 6), while positive beta values were shown in non-RH subjects (Figure 6). These findings suggest that the effect of rs7794745 genotype on voice-specific brain activity is opposite by the difference of handedness. A previous study has demonstrated that two SNPs in FOXP2 gene, which has been shown to be related to speech development, affect cognitive performance during dichotic listening task (Ocklenburg et al., 2013). Further, a recent neuroimaging study has demonstrated that some SNPs in *FOXP2* gene are associated with the determination of language dominance in the frontotemporal cortices during reading task (Pinel et al., 2012). However, to our knowledge, no study has as yet investigated the genetic effects on handedness and brain activity in auditory processing. Our results demonstrated an interaction effect among *CNTNAP2*, handedness, and cerebral response to human voice perception, although they showed no significant difference between handedness and brain activity in language processing. These findings suggest that the allele of rs7794745 affects cerebral response and laterality to human voice perception in MFG-STG in healthy individuals. A recent research has shown that the allele of rs7794745 affects patients with ASD (Li et al., 2010). Further, studies of patients with autism and schizophrenia have demonstrated impairment of voice-specific brain activity at the right STG (Ocklenburg et al., 2013). Taking this into account, in the evaluation of voice-specific response, this suggests that the difference of rs7794745 allele would be an important factor, as well as handedness.

Previous neuroimaging studies have shown that language dominance is affected by the difference of handedness (Pujol et al., 1999; Szaflarski et al., 2002). Especially, since language dominance is impacted by a family history of non-RH subjects (Szaflarski et al., 2002; Liu et al., 2009), genetic factor language-related gene, such as *CNTNAP2*, could associate with reduced language dominance or reversed language dominance. In our study, interaction effect between *CNTNAP2* genotype and handedness was not significantly observed in brain activity of language processing. These results suggest that rs7794745 and rs2710102 genotypes do not influence brain activity of language processing. On the other hand, our current findings, rs7794745 genotype affects voice-specific brain activity by the difference of handedness, suggest that cerebral response to human voice perception is genetically more susceptible than cerebral response to language processing. Regarding as voice-specific response in RH subjects, the group of genotype A/A in rs7794745 was observed at bilateral STG, whereas no bilateral STG activation was observed in the group of genotype A/T. In addition, concerning voice specific response in non-RH subjects, genotype A/A in rs7794745 was not shown at bilateral STG. These results may reflect that the group of rs7794745 genotype A/A can more easily respond to cerebral response to human voice perception than the rs7794745 genotype A/T group.

There are some limitations to the present study. First, although the allele effect on *CNTNAP2* was demonstrated in brain activity by passive listening task in our study, it is unclear whether the same genetic effect can be observed in any language processing. By each language processing task, the influence of *CNTNAP2* allele on brain activity could be different. Second, in our present study, behavior performance was not significantly different. Since behavior performance in every subject was extremely high, by the ceiling effect, it may be difficult to conclude whether the allele of *CNTNAP2* affecting brain activity is associated with any differences in language abilities. In behavioral performance, it was a very small effect size. These results may also be related to the ceiling effect due to the high performance of most subjects. Third, in our current study, we collected healthy volunteers without any psychiatric diseases, history of head injury, neurological disorder, alcohol, or drug dependence on the basis of interviews. However, a family history of these risks was not considered. Further, the subjects did not undergo verbal IQ and personality trait tests. Although educational levels of all subjects were more than 12 years, we should have considered the possibility that some of the subjects included a language impairment despite their high educational attainment.

## Conclusion

We investigated the influence of *CNTNAP2* on voice-specific brain activity, language-related brain activity, and handedness. Our results indicated that rs7794745 A/T in *CNTNAP2* is associated with a decrease in cerebral activation at R MFG and bilateral STG in human voice perception. This finding suggests that *CNTNAP2* polymorphisms could have a direct impact on right hemispheric activity for vocal communication as an endophenotype in healthy individuals. In addition, A/T in rs7794745 influences the activation of R MFG in language processing. This genotype may be related to a change in frontal activity in language processing of healthy subjects. Finally, our results demonstrated that rs7794745 has some influence on the difference in voice-specific brain activity between RH and non-RH subjects. These results suggest that *CNTNAP2* could be an important factor in the neural development related to vocal communication in both RH and non-RH subjects.

### Conflict of interest statement

The authors declare that the research was conducted in the absence of any commercial or financial relationships that could be construed as a potential conflict of interest.

## Appendix 1: contents of the sentences

We used the following sentences in the task.

1. Ms. Keiko Ueda, who lives in Kitakyushu city and works as a licensed cook at a company cafeteria, notified the police near the station that 56,000 yen was stolen when she was mugged at Odouri last night.
2. Last night, when Mr. Ichiro Sato was driving a 10-ton truck full of eggs along the road to Yokohama, near the mouth of the Tama River the axle of the truck broke, and the truck slipped off the road and was buried in a ditch.
3. These days, “Casual Day,” during which businessmen work in plain clothes with no tie, have been established, but the apparel business has developed and is marketing a “Dressed-Up Monday Campaign” that advertises “Let's be smartly dressed in a suit every Monday.”
4. Today, the designs of Northern Europe have become increasingly popular, and a cultural event showing a collection of Swedish designs, music and images, etc., called “Swedish style 2001,” will be held at various locations in Tokyo.

## Appendix 2: contents of the questions in the pilot study

### Appendix Questionnaire

Please answer the following question after listening to 2 sounds.

As what did you recognize these sounds? Please circle the appropriate one.

1. Human voice
2. Animal sound
3. Machine sound
4. Environmental sound

If you circled no. 1, please answer these questions.

As what did you recognize the first sound?

1. Male voice
2. Female voice

As what did you recognize the second sound?

1. Male voice
2. Female voice

Did you recognize these sounds as having intonation?

Yes No

Did you recognize a message from these sounds?

Yes No
