# Supplementary material for: Interaction effect between handedness and CNTNAP2 polymorphism (rs7794745 genotype) on voice-specific frontotemporal activity in healthy individuals: an fMRI study
Source: Front Behav Neurosci. 2015 Apr 20;9:87. doi: 10.3389/fnbeh.2015.00087 (PMC4403548; doi:10.3389/fnbeh.2015.00087)
Supplement: Supplementary file 1 [file Table1.DOCX]

**Supplemental Table 1**
